# Photodynamic Therapy in Cancer: Mechanisms, Photosensitizer Technology, Vitamin Modulation, and Rational Combination Design

**DOI:** 10.3390/biomedicines14091889

**Published:** 2026-08-24

**Authors:** Ilaf Naser, Dorota Bartusik-Aebisher, Barbara Smolak, Klaudia Dynarowicz, Edward Kowalczyk, Wiesław Guz, David Aebisher, Gabriela Henrykowska

**Affiliations:** 1 English Division Science Club, Faculty of Medicine, University of Rzeszów, 35-310 Rzeszów, Poland; in124003@stud.ur.edu.pl; 2 Department of Biochemistry and General Chemistry, Faculty of Medicine, University of Rzeszów, 35-310 Rzeszów, Poland; 3 Department of Diagnostic Imaging and Nuclear Medicine, Faculty of Medicine, University of Rzeszów, 35-310 Rzeszów, Poland; bsmolak@ur.edu.pl (B.S.);; 4 Department of Pharmacology and Toxicology, Faculty of Medicine, Medical University of Lodz, T. Kosciuszki 4, 90-419 Lodz, Poland; 5 Department of Photomedicine and Physical Chemistry, Faculty of Medicine, University of Rzeszów, 35-310 Rzeszów, Poland; 6 Department of Epidemiology and Public Health, Faculty of Medicine, Medical University of Lodz, T. Kosciuszki 4, 90-419 Lodz, Poland

**Keywords:** photodynamic therapy, photosensitizers, vitamin A, retinoids, vitamin D, reactive oxygen species, protoporphyrin IX, nanotechnology, immunogenic cell death, combination therapy

## Abstract

Photodynamic therapy (PDT) is a minimally invasive anticancer modality based on the interaction of a photosensitizer, light of an appropriate wavelength, and molecular oxygen, resulting in reactive oxygen species (ROS)-mediated tumor injury. This review discusses PDT as a mechanism-dependent therapeutic platform and evaluates how photosensitizers, vitamin A, vitamin D, nanotechnology, and combination strategies may influence its efficacy. The article synthesizes mechanistic and translational evidence concerning photosensitizer activation, ROS generation, tumor cell death, vascular shutdown, immunogenic cell death, and clinically relevant PDT applications. Particular attention is given to the divergent roles of vitamin A and vitamin D. Mechanistic and limited experimental evidence indicates that the effects of vitamin A-related compounds on PDT are heterogeneous and context-dependent. Selected compounds may attenuate PDT through antioxidant or cytoprotective mechanisms, whereas enhancement has also been reported for specific retinoid–photosensitizer combinations; however, clinical evidence remains limited. In contrast, vitamin D may enhance ALA/MAL-PDT by increasing intracellular protoporphyrin IX accumulation through modulation of the heme biosynthetic pathway, especially in non-melanoma skin cancer contexts. Nanocarriers, targeted photosensitizers, oxygen-modulating platforms, chemotherapy, and immunotherapy may further improve PDT when selected according to mechanistic compatibility. Overall, PDT combination design should be guided by whether adjunctive agents support or undermine photosensitizer accumulation, oxygen availability, ROS-mediated cytotoxicity, and immune activation.

## 1. Introduction

Photodynamic therapy (PDT) is a clinically established, minimally invasive anticancer modality based on the selective destruction of pathological tissue through light-induced oxidative damage. Unlike systemic chemotherapy, which exposes the entire organism to cytotoxic agents, PDT is spatially restricted: its effect occurs primarily where the photosensitizer has accumulated and where light is delivered. This gives PDT an important therapeutic advantage in superficial, localized, or anatomically accessible malignancies, where high local efficacy can be combined with relatively limited systemic toxicity [1,2].

In oncology, PDT has been used in the management of non-melanoma skin cancers, actinic keratoses, Bowen’s disease, superficial basal cell carcinoma, selected esophageal and endobronchial tumors, cholangiocarcinoma, and other lesions accessible to direct or endoscopic light delivery. Its clinical value results not only from direct tumor cell destruction but also from vascular injury within the tumor bed and the potential activation of antitumor immunity. Therefore, PDT should not be regarded merely as a local ablative technique. It is increasingly understood as a biologically complex treatment platform capable of interacting with tumor metabolism, vascular biology, immune signaling, and pharmacological modulation [3,4,5].

The renewed interest in PDT is also connected with the development of improved photosensitizers, nanotechnology-based delivery systems, oxygen-modulating strategies, and combination approaches with chemotherapy or immunotherapy. These advances aim to overcome the classical limitations of PDT, including insufficient light penetration, heterogeneous photosensitizer uptake, tumor hypoxia, and incomplete tumor selectivity. As a result, PDT is increasingly discussed not only as an independent therapeutic method but also as part of rational multimodal oncology [6,7].

The therapeutic mechanism of PDT depends on three indispensable components: a photosensitizer, light of an appropriate wavelength, and molecular oxygen within the treated tissue. None of these elements is sufficient alone. The photosensitizer must first accumulate in the tumor or premalignant lesion. The tissue must then be irradiated with light corresponding to the absorption spectrum of the photosensitizer. Finally, oxygen must be available in the local microenvironment to permit the generation of reactive oxygen species (ROS) [1,8].

After light activation, the photosensitizer enters an excited state and transfers energy or electrons to surrounding molecules and oxygen. This process leads to the formation of highly reactive species, including singlet oxygen, superoxide anion, hydroxyl radicals, and hydrogen peroxide. These molecules damage lipids, proteins, nucleic acids, mitochondria, lysosomes, cell membranes, and tumor-associated vasculature. Depending on the photosensitizer localization and the intensity of oxidative injury, PDT may induce apoptosis, necrosis, autophagy, acute vascular injury and vessel occlusion, or immunogenic cell death [4,5,8].

**Figure 1:**
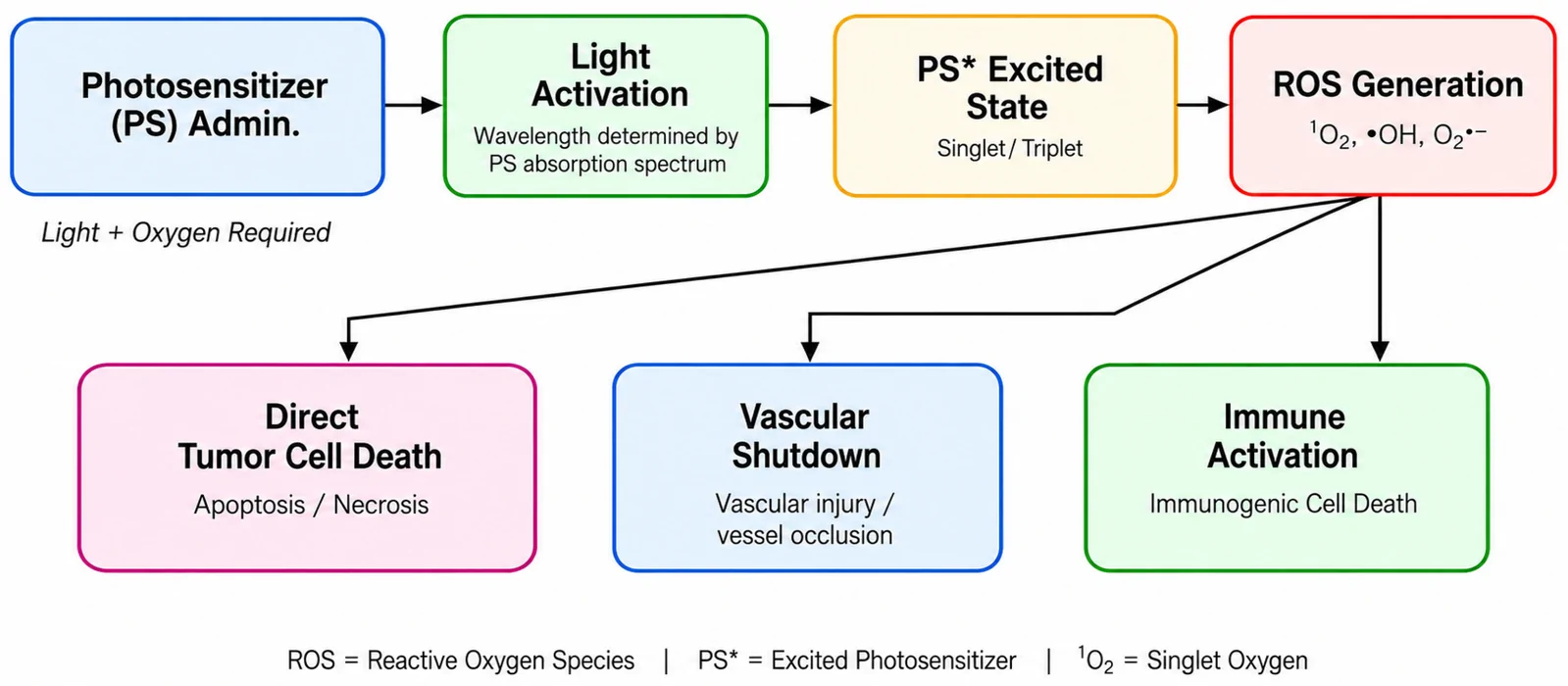
Photodynamic Therapy: Mechanism of Action. Light activation of the photosensitizer (PS) at a wavelength determined by its absorption spectrum generates an excited state (PS) that reacts with tissue oxygen to produce reactive oxygen species (ROS), including singlet oxygen (^1^O_2_), hydroxyl radicals (•OH), and superoxide (O_2_•^−^). These drive three complementary antitumor mechanisms: direct tumor cell death, vascular shutdown through vascular injury and vessel occlusion, and immune activation.

This dependence on ROS is central to the entire logic of PDT. The treatment is effective precisely because it generates oxidative stress at a level that exceeds the adaptive capacity of tumor cells. Therefore, all agents administered before or during PDT should be evaluated in relation to this mechanism. Compounds that enhance photosensitizer accumulation, improve oxygen availability, or amplify tumor-specific oxidative damage may improve PDT efficacy. Conversely, compounds that neutralize ROS or strengthen cellular antioxidant defenses may weaken treatment response. The general mechanism of PDT, including light-induced photosensitizer activation, ROS generation, and the resulting direct cytotoxic, vascular, and immune-mediated antitumor effects, is schematically presented in Figure 1 [1,4,8,9,10,11].

The biological outcome of PDT may be influenced by compounds that modify oxidative stress, cell survival pathways, photosensitizer metabolism, tumor oxygenation, or immune activation. This is particularly important in oncology because many patients use dietary supplements, vitamins, antioxidants, herbal preparations, or other biologically active substances during treatment. Such compounds are often perceived as generally protective or supportive, but their effect may be highly context-dependent [11,12,13].

Vitamin A and vitamin D illustrate this principle particularly clearly. Both are fat-soluble vitamins involved in the regulation of differentiation, proliferation, apoptosis, and tissue homeostasis. However, their interactions with PDT differ substantially.

Because vitamin A-related compounds differ substantially in their biological and pharmacological properties, their potential interactions with PDT cannot be generalized across all compounds or treatment protocols [13,14].

Vitamin D, by contrast, may support ALA/MAL-based PDT through a different mechanism. In this setting, vitamin D can increase intracellular accumulation of protoporphyrin IX (PpIX), the endogenous photosensitizer generated through the heme biosynthetic pathway after administration of 5-aminolevulinic acid or methyl aminolevulinate. By increasing photosensitizer loading in malignant or dysplastic cells, vitamin D may enhance the photodynamic response rather than suppress it. This contrast demonstrates that the relevance of a biologically active compound in PDT cannot be judged solely by its general anticancer potential. It must be assessed according to mechanistic compatibility with PDT-induced oxidative cytotoxicity [15,16].

This mechanism-based perspective is also relevant to other potential PDT modulators, including cytotoxic drugs, immunotherapeutic agents, nanocarriers, natural photosensitizers, oxygen-generating platforms, and tumor-targeting systems. Available experimental evidence concerning vitamin A-related compounds remains limited and context-dependent, indicating that their effects on PDT may vary according to the specific compound, dose, photosensitizer, tumor model, and experimental conditions [17,18].

The aim of this article is to discuss photodynamic therapy as an ROS-dependent anticancer strategy and to evaluate the role of photosensitizers, vitamin A, vitamin D, and selected combination approaches in shaping its therapeutic potential. Particular emphasis is placed on the mechanistic compatibility of adjunctive agents with PDT. This approach is essential because agents with superficially similar anticancer relevance may have opposite effects when placed in the specific biological context of photodynamic therapy.

The article first presents the mechanisms of PDT, including its photophysical and photochemical basis, Type I and Type II reactions, ROS generation, tumor cell damage, vascular effects, and immunogenic cell death. It then discusses photosensitizer technology, including first-, second-, and third-generation agents, natural photosensitizers, and the limitations of classical photosensitizing compounds [19,20].

A separate section is devoted to vitamin D as a rational neoadjuvant for ALA/MAL-PDT, particularly through its influence on PpIX accumulation. The article also reviews clinical applications of PDT in oncology, nanotechnology-based delivery systems, and rational combination strategies involving chemotherapy, radiotherapy, and immunotherapy. Finally, it addresses the practical significance of supplementation during cancer therapy, limitations of the current evidence base, and future directions for PDT development [19,20,21].

## 2. Materials and Methods

This narrative review was undertaken to provide a comprehensive overview of the current evidence concerning photodynamic therapy (PDT) in cancer, with particular emphasis on its molecular mechanisms, advances in photosensitizer technology, the influence of vitamins A and D on PDT efficacy, and the rationale for combination therapeutic approaches. Relevant literature was identified through searches of the PubMed, Scopus, and Web of Science databases. The searches covered publications from database inception through 28 July 2026. Priority was given to peer-reviewed articles published in English, including original research papers, clinical studies, systematic reviews, meta-analyses, and authoritative review articles. Earlier landmark publications were also considered when necessary to describe fundamental concepts and the historical development of PDT. The search strategy included combinations of keywords related to the main topics of this review, including photodynamic therapy, photosensitizers, 5-aminolevulinic acid, methyl aminolevulinate, reactive oxygen species, vitamin A, retinoids, vitamin D, calcitriol, protoporphyrin IX, nanotechnology, drug delivery, immunotherapy, combination therapy, tumor microenvironment, and cancer.

The literature was selected according to its scientific relevance, methodological quality, and contribution to understanding the biological mechanisms, translational aspects, and clinical applications of PDT. Methodological quality was assessed descriptively according to study design, appropriateness of the experimental or clinical model, clarity of the intervention and comparator, adequacy of outcome reporting, and consistency between the reported results and conclusions. Evidence categories used in the tables were assigned according to the highest level of evidence available for each intervention. Reproducible experimental studies were classified as preclinical evidence, observational studies and small clinical studies as limited clinical evidence, and randomized controlled trials or consistently supported clinical findings as clinical evidence. These categories were used to structure the narrative synthesis and were not intended to constitute a formal systematic evidence-grading framework. Particular emphasis was placed on publications that provided mechanistic insight or clinical evidence supporting the optimization of PDT and rational combination strategies. The final review incorporates 103 references considered representative of the current state of knowledge.

## 3. Mechanisms of Action of Photodynamic Therapy

Photodynamic therapy (PDT) is based on a coordinated sequence of photophysical, photochemical, cellular, vascular, and immunological events. Its therapeutic activity requires the simultaneous presence of three essential components: a photosensitizer, light of an appropriate wavelength, and molecular oxygen within the treated tissue. The photosensitizer itself is not cytotoxic in the absence of light, and light alone is insufficient to produce a therapeutic response. Only when the photosensitizer absorbs photons in an oxygenated environment does the photodynamic reaction begin, leading to the generation of reactive oxygen species (ROS) and subsequent tumor destruction. This three-component dependency explains both the selectivity and the limitations of PDT. The treatment can be spatially controlled by restricting light exposure to the tumor area, but its efficacy remains dependent on adequate photosensitizer accumulation, sufficient light penetration, and oxygen availability in the tumor microenvironment [20,22,23].

The photochemical mechanism begins when a photosensitizer in its ground singlet state absorbs light energy and is promoted to an excited singlet state. This excited state is unstable and short-lived. It may return to the ground state by emitting energy, or it may undergo intersystem crossing to form a longer-lived excited triplet state. The triplet state is the key biologically productive state in PDT, because it allows the activated photosensitizer to interact with surrounding biomolecules and molecular oxygen. Through these interactions, the photosensitizer initiates oxidative reactions that ultimately damage malignant cells and tumor-associated structures. This photophysical sequence—photon absorption, excited singlet-state formation, intersystem crossing, and triplet-state reactivity—forms the core chemical basis of PDT-induced cytotoxicity [24].

Once the photosensitizer reaches the excited triplet state, two principal photochemical pathways may occur: Type I and Type II reactions. In Type I reactions, the activated photosensitizer transfers electrons or hydrogen atoms to nearby biological substrates. This process generates radical intermediates, including superoxide anion, hydroxyl radicals, and hydrogen peroxide. These molecules initiate oxidative chain reactions that damage lipids, proteins, enzymes, membranes, and nucleic acids. In Type II reactions, the triplet-state photosensitizer transfers energy directly to ground-state molecular oxygen, producing singlet oxygen. Singlet oxygen is highly reactive and short-lived and acts close to the site where it is generated. For this reason, the intracellular localization of the photosensitizer strongly influences the biological outcome of PDT. Both Type I and Type II mechanisms may occur simultaneously, although singlet oxygen generated through the Type II pathway is often considered the dominant cytotoxic mediator in many PDT systems [25,26,27].

PDT-induced tumor cell death is not uniform. The dominant mode of death depends largely on where the photosensitizer accumulates inside the cell and on the intensity of oxidative damage. Mitochondria-localized photosensitizers tend to induce apoptosis by damaging mitochondrial membranes, disrupting mitochondrial membrane potential, releasing cytochrome c, and activating caspase-dependent signaling pathways. Lysosome-associated photosensitizers may cause lysosomal membrane permeabilization, release of hydrolytic enzymes, autophagy-associated damage, or necrotic cell death. Photosensitizers localized in or near the plasma membrane can produce rapid lipid peroxidation, membrane rupture, ionic imbalance, and acute necrosis. Therefore, PDT can eliminate cancer cells through apoptosis, necrosis, autophagy-related mechanisms, or mixed forms of cell death depending on drug localization, light dose, oxygen status, and tumor biology [26,28].

In addition to direct cytotoxicity, PDT exerts important vascular effects. Oxidative damage to endothelial cells within tumor vessels may induce vasoconstriction, platelet aggregation, increased vascular permeability, blood-flow stasis, and vessel occlusion. This vascular shutdown deprives residual malignant cells of oxygen and nutrients, thereby amplifying tumor destruction beyond the cells directly damaged during light exposure. In some tumors, vascular injury may contribute substantially to local tumor control [23,29].

A further mechanism of PDT is activation of antitumor immunity. Photodynamic injury can induce immunogenic cell death, during which dying tumor cells expose or release danger signals and tumor-associated antigens. These include calreticulin exposure on the cell surface, extracellular ATP release, HMGB1 release, and liberation of antigenic tumor material. Together, these signals promote dendritic cell recruitment, antigen uptake, dendritic cell maturation, antigen presentation, and activation of tumor-specific cytotoxic T lymphocytes. As a result, PDT may convert the treated tumor into an in situ source of tumor antigens capable of stimulating local and systemic immune responses. This immunological mechanism provides the rationale for combining PDT with immunotherapy, particularly immune checkpoint inhibitors targeting PD-1, PD-L1, or CTLA-4. Preclinical evidence suggests that PDT-induced immunogenic cell death may enhance systemic antitumor immunity and may contribute to regression of distant, non-irradiated lesions when combined with checkpoint blockade [30,31,32].

The mechanisms of PDT are therefore interconnected rather than isolated. Light activation of the photosensitizer initiates photochemical ROS production; ROS damage tumor cells and tumor vasculature; vascular shutdown intensifies local tumor injury; and immunogenic cell death may transform local phototoxicity into a broader antitumor immune response. This integrated mechanism also explains why PDT is highly sensitive to agents that modify oxidative stress, photosensitizer accumulation, oxygen availability, or antioxidant defenses. Compounds that increase photosensitizer loading, improve oxygenation, or amplify tumor-specific oxidative damage may enhance PDT. Conversely, agents that scavenge ROS, stabilize cellular membranes, or activate antioxidant survival pathways may weaken PDT efficacy. This principle is central to the later evaluation of vitamin A derivatives and vitamin D. Current evidence indicates that the effects of vitamin A derivatives on PDT are context-dependent and cannot be generalized across different retinoid species or PDT protocols. By contrast, vitamin D has demonstrated promising adjuvant effects in ALA- and MAL-based PDT by enhancing intracellular protoporphyrin IX accumulation, particularly in superficial keratinocytic lesions and non-melanoma skin cancers [32,33,34].

### Optical Dosimetry in Photodynamic Therapy

The therapeutic efficacy of photodynamic therapy depends not only on the biological properties of the photosensitizer and tissue oxygenation but also on the precise delivery of light to the target tissue. Optical dosimetry refers to the measurement and optimization of light parameters necessary to activate the photosensitizer and generate sufficient reactive oxygen species while minimizing damage to surrounding healthy tissue [35]. Therefore, dosimetry has become an essential element of modern PDT protocols, particularly in the context of individualized treatment planning [36].

The most important dosimetric parameter is the excitation wavelength of the light, which must match the absorption spectrum of the selected photosensitizer. In clinical oncology, most photosensitizers are activated with red light with a wavelength of approximately 630–690 nm, as this spectral range represents a compromise between effective photosensitizer excitation and relatively deep tissue penetration [37]. Longer wavelengths penetrate tissue more effectively due to reduced absorption by hemoglobin and melanin, while shorter wavelengths are typically limited to superficial lesions, despite stronger absorption by some photosensitizers [38].

Another critical parameter is light fluence (J/cm^2^), representing the total optical energy delivered per unit area. Fluence determines the cumulative photodynamic dose received by the tissue and directly influences the amount of ROS generated during irradiation [39]. Equally important is the fluence rate, or irradiance intensity (mW/cm^2^), which determines the rate of energy delivery. Excessively high irradiance can rapidly consume available oxygen in the irradiated tissue, causing transient hypoxia and reduced ROS production despite the same total light dose. Therefore, protocols with lower irradiance intensity or fractionated irradiation can improve treatment efficacy by enabling oxygen replenishment during irradiation [40]. Light propagation in biological tissues is further influenced by optical scattering and absorption. Tissue composition, vascularization, pigmentation, and anatomical location modify the distribution of photons, causing the effective light dose to differ significantly from the dose incident on the surface. These factors contribute to the significant heterogeneity of PDT responses and partially explain why standardized illumination protocols do not always achieve identical clinical outcomes for different tumor types [41].

PDT dosimetry is conventionally classified as explicit, implicit, or direct [35,42]. Explicit dosimetry quantifies the principal treatment inputs, including the delivered light dose, photosensitizer concentration, and tissue oxygenation. Implicit dosimetry uses treatment-induced changes, particularly photosensitizer fluorescence and photobleaching, as surrogate indicators of the effective photodynamic dose. Direct dosimetry aims to measure the cytotoxic photoproduct itself, most notably through singlet oxygen luminescence detection in experimental systems. These approaches provide complementary information and may facilitate individualized treatment optimization. Optical dosimetry should be considered one of the three fundamental pillars of PDT, alongside photosensitizer pharmacology and tissue oxygenation. Further development of dosimetric strategies will improve treatment reproducibility and maximize therapeutic efficacy. The principal optical parameters used in PDT dosimetry and their clinical significance are summarized in Table 1. Together, these variables determine the effective photodynamic dose and should be optimized according to the characteristics of the photosensitizer, target tissue, and clinical indication.

## 4. Photosensitizers in Photodynamic Therapy

Photosensitizers are the central pharmacological component of photodynamic therapy, because they determine whether absorbed light energy can be converted into biologically destructive oxidative stress. The therapeutic effect of PDT depends not only on the presence of light and oxygen but also on the chemical, optical, and biological properties of the photosensitizing compound. An effective photosensitizer must absorb light efficiently, reach the target tissue in sufficient concentration, generate reactive oxygen species after irradiation, and remain minimally toxic in the absence of light. Therefore, the development of photosensitizers has been one of the most important determinants of PDT progress in oncology [43,44,45].

An ideal photosensitizer should meet several criteria. First, it should have strong absorption within the therapeutic optical window, generally in the red or near-infrared range, where tissue penetration is superior to shorter wavelengths. Second, it should generate singlet oxygen and other ROS with high quantum efficiency after irradiation. Third, it should accumulate preferentially in malignant or dysplastic tissue while clearing rapidly from normal tissues to reduce prolonged photosensitivity. Fourth, it should have low dark toxicity, meaning that it should be biologically inactive or minimally toxic before light activation. Additional desirable properties include chemical purity, water solubility, predictable pharmacokinetics, metabolic stability before irradiation, rapid elimination after treatment, and compatibility with topical, systemic, endoscopic, or nanotechnology-based delivery [45,46,47].

The first clinically important group of photosensitizers consisted of hematoporphyrin derivatives, with porfimer sodium, also known as Photofrin, as the best-known example. These first-generation agents established PDT as a clinically applicable cancer treatment and remain relevant in selected indications, particularly esophageal and endobronchial malignancies. However, their limitations are substantial. They are chemically heterogeneous mixtures rather than single well-defined compounds, show relatively weak absorption in the optimal red-light range, and have only moderate tumor selectivity. One of their most clinically inconvenient drawbacks is prolonged cutaneous photosensitivity, which may persist for several weeks after administration. These disadvantages created the need for photosensitizers with improved absorption spectra, better pharmacokinetics, higher selectivity, and shorter periods of post-treatment light sensitivity [44,48].

Second-generation photosensitizers were developed to address these limitations. This group includes chemically defined porphyrins, chlorins, bacteriochlorins, phthalocyanines, temoporfin, and prodrug-based systems such as 5-aminolevulinic acid and methyl aminolevulinate. Compared with first-generation compounds, many second-generation agents absorb light at longer wavelengths, often within the 600–800 nm range, which improves tissue penetration and makes them more suitable for clinical irradiation. They also tend to have greater chemical purity, stronger photodynamic activity, and more predictable pharmacological behavior. Temoporfin and other chlorin-based compounds, for example, exhibit strong absorption in the red spectrum and high photodynamic potency, while phthalocyanines are characterized by intense absorption at longer wavelengths and favorable photophysical properties [49,50].

ALA and MAL occupy a particularly important position among second-generation strategies because they are not conventional photosensitizers in the strict sense. Instead, they act as precursors in the heme biosynthetic pathway and lead to intracellular accumulation of protoporphyrin IX (PpIX), an endogenous photosensitizer. This mechanism is highly relevant in dermatological oncology and photodiagnosis, especially in actinic keratoses, Bowen’s disease, and superficial basal cell carcinoma. Tumor and dysplastic cells may accumulate more PpIX than surrounding normal tissue because of altered heme metabolism, including increased synthesis and reduced conversion of PpIX to heme. This selective accumulation forms the biochemical basis for ALA/MAL-PDT and is also essential for understanding why vitamin D may enhance this form of treatment by increasing intracellular PpIX levels [51,52].

Third-generation photosensitizers represent a shift from simple light-sensitive molecules toward engineered therapeutic systems. These agents are designed to improve tumor selectivity, enhance delivery, reduce systemic toxicity, and enable combination treatment. Third-generation approaches may involve conjugation of photosensitizers with targeting ligands such as antibodies, peptides, folic acid, or aptamers. These ligands can direct the photosensitizer toward tumor-associated receptors or microenvironmental features. Another major third-generation strategy is incorporation into nanocarriers, including liposomes, polymeric nanoparticles, gold nanoparticles, micelles, and other multifunctional platforms. These systems can improve solubility, prevent aggregation, increase tumor accumulation, control drug release, and allow co-delivery with chemotherapeutic agents, immunomodulators, or oxygen-generating compounds [53,54].

Natural and plant-derived photosensitizers have also attracted increasing attention as potentially biocompatible alternatives or complements to synthetic photosensitizers. Hypericin, derived from *Hypericum perforatum*, is a potent natural photosensitizer capable of generating singlet oxygen and showing tumor-selective properties. Curcumin, derived from *Curcuma longa*, exhibits photoactivity mainly in the blue-light range and has additional anti-inflammatory and biological effects, although its clinical use is limited by poor aqueous solubility and restricted tissue penetration. Chlorophyll and pheophorbide derivatives are attractive because they are structurally related to porphyrin-like systems and may absorb longer wavelengths, making them relevant for PDT development. Other natural compounds, including riboflavin and berberine, also demonstrate photosensitizing potential under appropriate irradiation conditions [55].

Despite these advances, classical photosensitizers still face important limitations. Many are hydrophobic and tend to aggregate in aqueous environments, which can reduce photodynamic efficiency through self-quenching and poor tissue distribution. Some have insufficient selectivity between tumor and normal tissue, causing off-target phototoxicity. Others persist in the skin or other tissues for prolonged periods, increasing the risk of photosensitivity reactions after treatment. Heterogeneous tumor uptake may lead to incomplete treatment response, especially in larger or poorly vascularized tumors. In addition, even highly efficient photosensitizers require oxygen, so hypoxic tumor regions may remain resistant to PDT. Limited light penetration further restricts the use of conventional PDT in deep-seated tumors [45,56].

**Figure 2:**
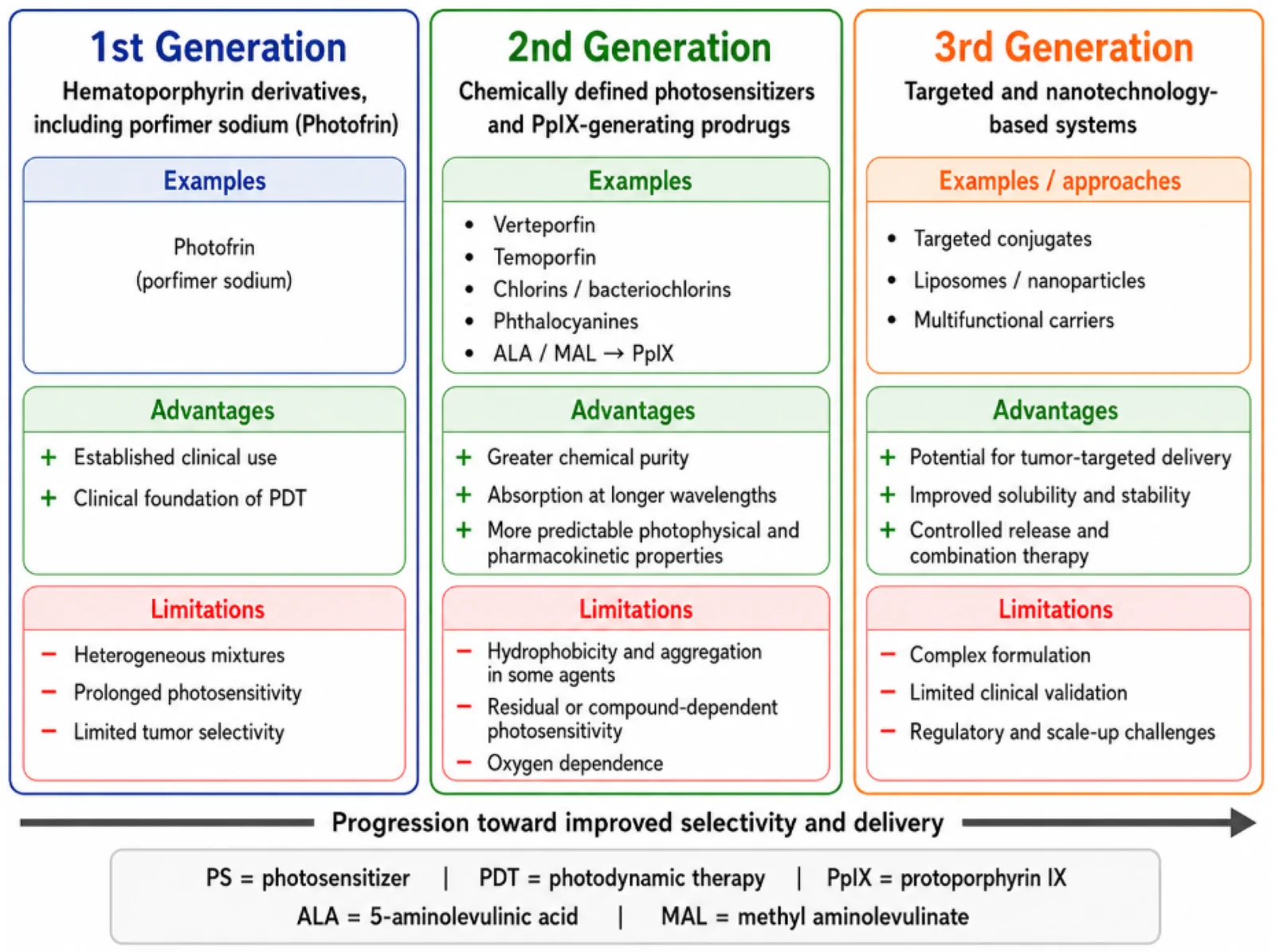
Classification and evolution of photosensitizers used in photodynamic therapy. First-generation photosensitizers are represented by hematoporphyrin derivatives, including porfimer sodium (Photofrin), which established the clinical basis of PDT but are limited by chemical heterogeneity, prolonged photosensitivity, and limited tumor selectivity. Second-generation agents include chemically defined photosensitizers, such as verteporfin, temoporfin, chlorins, bacteriochlorins, and phthalocyanines, as well as the PpIX-generating prodrugs ALA and MAL. These agents generally offer greater chemical purity, absorption at longer wavelengths, and improved pharmacokinetic and selectivity profiles, although hydrophobicity, aggregation, residual photosensitivity, and oxygen dependence remain important limitations. Third-generation systems incorporate targeted conjugates, liposomes, nanoparticles, and multifunctional carriers to improve tumor-directed delivery, solubility, stability, controlled release, and combination therapy, but their clinical translation is constrained by formulation complexity, limited clinical validation, and regulatory and scale-up challenges [43,44,51,55].

The development of newer photosensitizers is therefore directed toward overcoming these barriers. Current strategies focus on improving absorption within the therapeutic optical window, increasing tumor-specific accumulation, enhancing ROS generation, reducing dark toxicity, shortening systemic photosensitivity, and improving delivery to hypoxic or difficult-to-reach tumor regions. Nanotechnology and molecular targeting are particularly important in this context because they transform photosensitizers from passive light-activated compounds into components of rationally designed anticancer systems. The principal differences among first-, second-, and third-generation photosensitizers are summarized in Figure 2 [45,47,53,55].

## 5. Practical Significance of Supplementation During Cancer Therapy

### 5.1. Vitamin A in the Context of Anticancer Therapy

Vitamin A is a group of fat-soluble compounds with broad biological relevance for epithelial integrity, immune function, cellular differentiation, proliferation, and apoptosis. In biological systems, vitamin A comprises a group of structurally and functionally distinct compounds, including retinol, retinal, retinoic acid, retinyl esters, and provitamin A carotenoids such as β-carotene. Although these compounds are metabolically interconnected through enzymatic conversion pathways, they differ substantially in their biological functions, pharmacological properties, and mechanisms of action. Consequently, findings obtained for one vitamin A species should not be generalized to other retinoids or carotenoids. In the oncology setting, specific vitamin A derivatives have been shown to influence gene expression, cellular differentiation, cell-cycle progression, oxidative stress responses, and cell survival; however, these effects are highly dependent on the individual compound, dose, and biological context [57,58]. Retinol is the principal circulating form of vitamin A and is stored predominantly as retinyl esters, mainly retinyl palmitate, in the liver and other tissues. Retinol may be reversibly oxidized to retinal, which can subsequently be irreversibly converted to all-trans retinoic acid, the major transcriptionally active metabolite. Retinoic acid regulates gene expression primarily through activation of retinoic acid receptors (RARs) and retinoid X receptors (RXRs), whereas retinol functions mainly as a metabolic intermediate and plays an essential role in the visual cycle. Provitamin A carotenoids, particularly β-carotene, are dietary precursors of vitamin A but also possess antioxidant properties, including singlet oxygen quenching, that are independent of their enzymatic conversion to retinoids. These distinct biological activities are particularly relevant in the context of photodynamic therapy, where the effects of individual vitamin A species may differ depending on their specific mechanism of action, the photosensitizer used, and the experimental or clinical setting [57,58,59,60,61].

The biological effects of retinoic acid are mediated primarily through nuclear retinoid receptors: retinoic acid receptors and retinoid X receptors. These receptors function as ligand-activated transcription factors. After binding retinoic acid, they form receptor dimers and regulate the expression of genes involved in differentiation, proliferation arrest, apoptosis, epithelial maturation, immune regulation, and tissue homeostasis. Through these pathways, retinoids can modify the phenotype of malignant cells and influence tumor biology at the transcriptional level. This receptor-mediated mechanism explains why retinoids have been investigated as anticancer agents and why they are therapeutically useful in selected malignancies [58,59].

The clearest clinical example of beneficial retinoid activity is acute promyelocytic leukemia, where all-trans retinoic acid induces differentiation of malignant promyelocytes and forms a core component of highly effective treatment strategies. In this setting, the therapeutic effect of retinoids results not from direct oxidative cytotoxicity, but from restoration of differentiation and disruption of the leukemic maturation block. This distinction is crucial. Retinoids may have legitimate anticancer activity in contexts where differentiation, transcriptional regulation, or apoptosis induction is the therapeutic objective. However, this does not mean that they are automatically compatible with all cancer therapies. Their effect depends on the dominant mechanism of the treatment with which they are combined [58,62,63].

Retinoids may also influence apoptosis and cell survival pathways. Depending on cellular context, receptor expression, dose, and tumor type, retinoic acid can promote cell-cycle arrest, enhance differentiation, or induce programmed cell death. These effects have supported interest in retinoids as chemopreventive or therapeutic agents in selected cancer models. However, retinoid signaling is complex and context-dependent. In some cellular environments, retinoids may activate proapoptotic programs, while in others they may reinforce survival responses, increase stress tolerance, or induce adaptive antioxidant pathways. This context dependency is especially relevant when retinoids are considered alongside therapies such as PDT, radiotherapy, or chemotherapy, where oxidative injury contributes substantially to tumor cell killing [58,60,64,65].

A major issue in the PDT context is the antioxidant activity of vitamin A-related compounds. Carotenoids, including β-carotene, can quench singlet oxygen and neutralize lipid peroxyl radicals. Retinol may stabilize cellular and mitochondrial membranes, thereby reducing lipid peroxidation. Retinoic acid may also activate antioxidant response programs, including pathways associated with nuclear factor erythroid 2-related factor 2, which regulates genes involved in glutathione synthesis, superoxide dismutase activity, catalase expression, and other ROS-detoxifying systems. These effects can protect cells from oxidative injury. Under physiological conditions, such cytoprotection may be beneficial; however, in some PDT settings, it may reduce photodynamic injury, although this effect cannot be generalized across all vitamin A-related compounds or treatment protocols [61,66].

The cytoprotective properties of vitamin A are directly relevant to ROS-dependent cancer therapies. PDT requires the accumulation of reactive oxygen species at levels sufficient to overwhelm tumor cell defenses. If a specific vitamin A-related compound scavenges ROS, stabilizes membranes, reduces lipid peroxidation, or activates antioxidant transcriptional programs under a given treatment condition, it may increase the ability of tumor cells to survive photodynamic injury. Similar concerns apply to other therapies in which oxidative stress contributes to cytotoxicity, including radiotherapy and several chemotherapeutic classes. Therefore, vitamin A should not be evaluated only as a micronutrient or general anticancer compound, but also as a potential modifier of treatment-induced oxidative stress [61,65,67].

This distinction is clinically important because patients with cancer frequently use supplements, including multivitamins, β-carotene, retinol-containing preparations, and antioxidant formulations. These products may be taken with the intention of supporting health or reducing treatment toxicity, but they may also interfere with therapies that depend on oxidative damage. In the context of PDT, the potential concern relates not to normal dietary vitamin A intake but rather to high-dose pharmacological supplementation that may alter redox balance and cytoprotective signaling. Similar considerations may apply to antioxidant supplementation during radiotherapy or chemotherapy when ROS-mediated injury contributes to the therapeutic mechanism. However, current evidence remains insufficient to support general recommendations regarding the use or avoidance of vitamin A supplementation during anticancer therapy. Any decision concerning high-dose vitamin A supplementation should therefore be individualized and made in consultation with the treating oncology team [60,65,67].

Thus, vitamin A and retinoids occupy a complex position in oncology. They may be therapeutically valuable in differentiation-based treatment, biologically relevant in cancer prevention and epithelial regulation, and capable of modifying oxidative-stress-dependent treatment in a compound- and context-dependent manner. Their role in PDT must therefore be assessed according to mechanistic compatibility rather than general anticancer reputation. Because PDT depends on ROS generation, membrane oxidation, organelle damage, vascular injury, and immunogenic cell death, specific compounds that reinforce antioxidant and cytoprotective defenses may reduce treatment efficacy under particular experimental or clinical conditions, whereas other retinoid–PDT combinations may produce neutral or enhancing effects [58,60,61,64,65,66].

### 5.2. Potential Interactions Between Vitamin A Derivatives and PDT

Vitamin A-related compounds may modify PDT responses through several distinct mechanisms, but the direction and magnitude of these interactions cannot be generalized across the entire class. Retinoid receptor-mediated signaling, carotenoid-mediated singlet oxygen quenching, and the pharmacological effects of retinol or retinyl esters represent biologically distinct processes. These mechanistic pathways may influence PDT responses, but the available mechanistic evidence does not by itself establish a consistent direction or magnitude of effect on PDT-induced cytotoxicity [68,69,70,71].

The hypothesis that selected vitamin A-related compounds may attenuate PDT is based primarily on their antioxidant or cytoprotective properties. β-Carotene and other provitamin A carotenoids have been shown to quench singlet oxygen and scavenge lipid peroxyl radicals, whereas retinol has been reported to influence membrane stability and cellular redox homeostasis under certain experimental conditions. These distinct biological properties have led to the hypothesis that selected vitamin A species could modulate ROS-dependent photodynamic responses. However, the available evidence remains limited and heterogeneous, and the effects appear to depend on the specific vitamin A derivative, photosensitizer, dose, and experimental model. Retinoic acid may also activate antioxidant response pathways, including signaling associated with nuclear factor erythroid 2-related factor 2 (Nrf2), which regulates glutathione synthesis, superoxide dismutase, catalase, and other ROS-detoxifying systems. These effects could attenuate oxidative injury in specific experimental settings [70,71,72,73].

Potential attenuation of PDT may occur through several mechanisms. Direct ROS or singlet oxygen quenching may reduce the effective photodynamic oxidant burden, reinforcement of endogenous antioxidant systems may increase cellular stress tolerance, and membrane stabilization may limit lipid peroxidation and organelle injury. However, these mechanisms should not be attributed uniformly to all retinoids, carotenoids, or retinyl esters, and mechanistic plausibility alone does not establish a consistent antagonistic effect [68,69,70,71,72,73].

In the rhabdomyosarcoma study evaluating vitamin A-related compounds in combination with PDT, the interactions were analysed using the Chou–Talalay method. Importantly, the results differed according to the photosensitizing system: findings obtained with 5-ALA-PDT should be distinguished from those obtained with Photogem-PDT rather than interpreted as evidence of uniform vitamin A antagonism. The reported combination index values above 1 therefore apply to specific compound–photosensitizer combinations and experimental conditions, not to vitamin A-related compounds as a single pharmacological class [74].

Conversely, all-trans retinoic acid has also been reported to enhance fimaporfin-based PDT. This apparently opposite finding indicates that retinoid–PDT interactions may vary with the retinoid species, photosensitizer, dose, tumor model, treatment sequence, and experimental conditions. The available evidence therefore supports a context-dependent interpretation rather than a universal antagonistic relationship [34].

The clinical implications remain uncertain and should be interpreted cautiously. The available direct evidence derives predominantly from in vitro studies and includes both attenuating and enhancing interactions. Consequently, it does not support a general conclusion that vitamin A or its derivatives antagonize PDT. High-dose vitamin A, β-carotene, or retinoid-containing supplementation should therefore be evaluated individually according to the specific compound, dose, PDT protocol, tumor type, and clinical indication, in consultation with the treating oncology team. This concern is especially important because supplement use among cancer patients is common and may not always be disclosed during routine clinical assessment [75].

The same reasoning extends to other oxidative-stress-dependent cancer therapies. Radiotherapy induces DNA damage partly through water radiolysis and ROS generation. Several chemotherapeutic agents, including platinum compounds, alkylating agents, and anthracyclines, also involve oxidative stress as one component of their antitumor activity. If a specific vitamin A-related intervention strengthens antioxidant defenses or reduces ROS-mediated injury under the relevant treatment conditions, it could theoretically modify the efficacy of these therapies. Given the widespread use of dietary supplements among patients with cancer, clinicians should routinely inquire about supplement use during treatment. Decisions regarding high-dose vitamin A or β-carotene supplementation during PDT or other anticancer therapies should be individualized and made in consultation with the treating oncology team [74,75,76].

**Figure 3:**
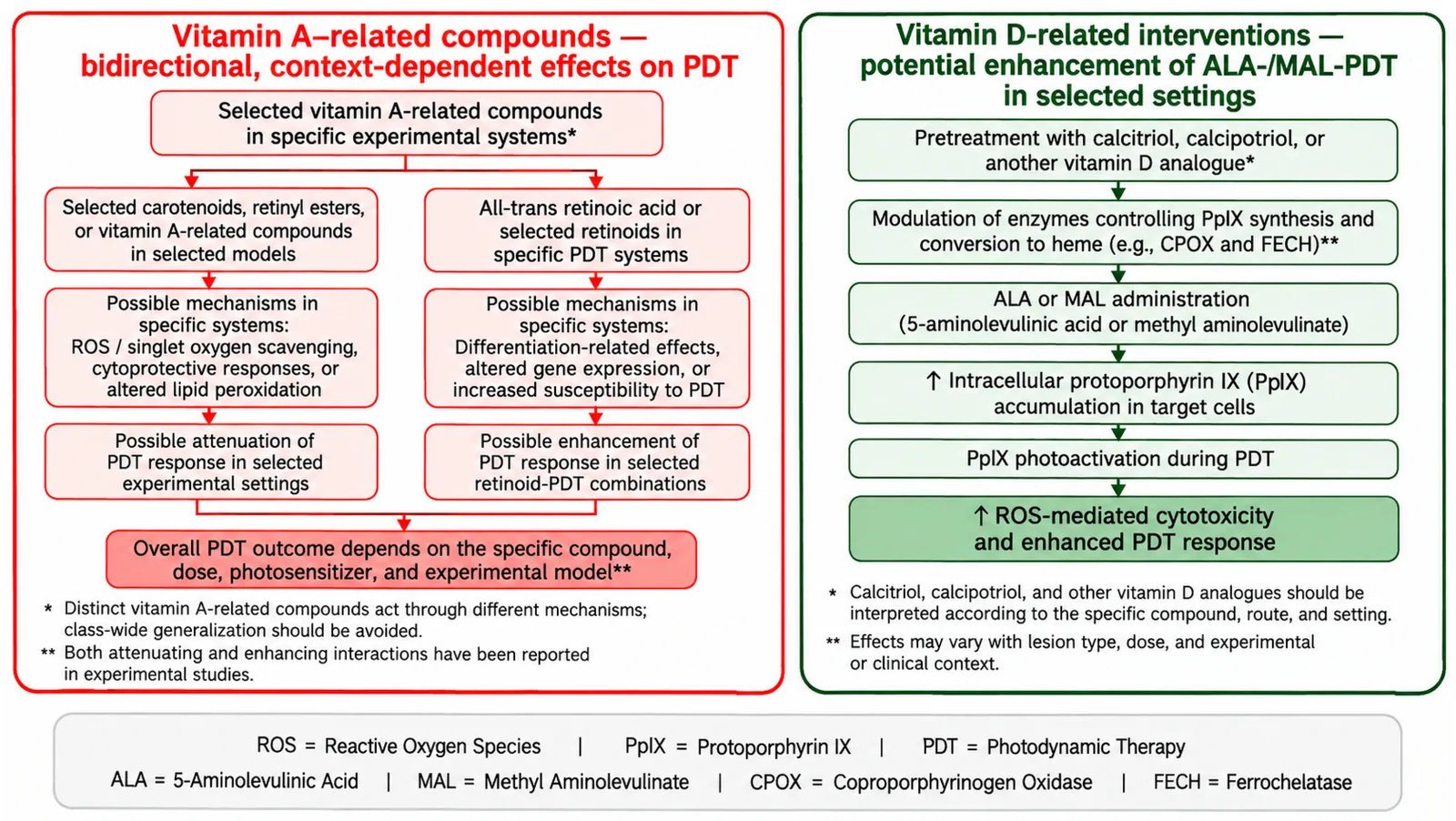
Context-dependent interactions of vitamin A-related compounds and vitamin D-related interventions with photodynamic therapy. (**Left panel**): specific retinoids, carotenoids, and other vitamin A-related compounds may attenuate, enhance, or have no measurable effect on PDT depending on the compound, dose, photosensitizer, tumor model, and experimental conditions. Antagonistic interactions, including combination index (CI) values above 1 [74], have been reported for specific vitamin A-related compound–PDT combinations in vitro, whereas enhancement has also been observed with all-trans retinoic acid in a fimaporfin-based PDT system [34]. (**Right panel**): calcitriol or vita-min D analogues may modulate the heme biosynthetic pathway and increase intracellular protoporphyrin IX (PpIX) accumulation before ALA- or MAL-based PDT. These effects should be interpreted separately according to the vitamin D compound, route of administration, lesion type, photosensitizer precursor, and study endpoint. Abbreviations: ROS, reactive oxygen species; PpIX, protoporphyrin IX; PDT, photodynamic therapy; CI, combination index; ALA, 5-aminolevulinic acid; MAL, methyl aminolevulinate.

Vitamin A-related compounds should therefore be evaluated separately from vitamin D in the PDT context. Although both groups may influence cancer biology, their effects depend on distinct molecular mechanisms and treatment conditions. Selected retinoids or carotenoids may attenuate PDT through antioxidant or cytoprotective mechanisms, whereas other retinoid–photosensitizer combinations may enhance photodynamic efficacy. Vitamin D-related interventions may enhance ALA- or MAL-based PDT by increasing PpIX accumulation, although this effect also depends on the specific vitamin D compound, route of administration, lesion type, and PDT protocol. These context-dependent interactions are summarized in Figure 3 [34,68,74,77,78].

### 5.3. Vitamin D as a Rational Neoadjuvant for ALA/MAL-PDT

Vitamin D-related interventions represent mechanistically distinct potential neoadjuvant strategies for 5-aminolevulinic acid- or methyl aminolevulinate-based photodynamic therapy. Unlike the heterogeneous and context-dependent interactions reported for vitamin A-related compounds, calcitriol and selected vitamin D analogues have been investigated primarily for their capacity to increase photosensitizer accumulation before ALA- or MAL-based PDT. In ALA/MAL-PDT, the therapeutic photosensitizer is not administered as a fully formed exogenous compound.

The biological activity of vitamin D is mediated primarily through the vitamin D receptor (VDR), a nuclear receptor that regulates transcriptional programs involved in differentiation, proliferation, apoptosis, immune modulation, and tissue homeostasis. The active hormonal form of vitamin D, calcitriol, binds to VDR and influences the expression of genes that can alter cellular phenotype and metabolic state. In cancer biology, this is relevant because differentiation status, metabolic activity, and enzyme expression can affect how tumor cells process ALA or MAL and how much PpIX they accumulate before illumination. Calcitriol does not enhance PDT through direct light activation but may act upstream of irradiation by modifying cellular differentiation and heme-pathway enzyme expression. Instead, its key contribution in ALA/MAL-PDT is upstream of light activation: it increases the intracellular pool of the photosensitizing metabolite that will later be activated by irradiation.

The heme biosynthetic pathway is central to this effect. ALA is the first committed precursor in heme synthesis. When exogenous ALA or MAL is administered, cells convert it through a sequence of enzymatic steps into PpIX. Under normal physiological conditions, PpIX is subsequently converted into heme by ferrochelatase, which inserts ferrous iron into the porphyrin ring. In many tumor and premalignant cells, this pathway is altered in a way that favors PpIX accumulation: upstream synthetic activity may be increased, while the final conversion of PpIX to heme may be relatively reduced. This imbalance allows PpIX to accumulate preferentially in abnormal tissue, creating the biochemical basis for selective ALA/MAL-PDT.

Vitamin D has been shown to enhance intracellular PpIX accumulation by modulating the heme biosynthetic pathway, including increased expression of coproporphyrinogen oxidase and reduced expression of ferrochelatase, thereby favoring PpIX accumulation prior to ALA-PDT. This mechanistic evidence primarily concerns calcitriol pretreatment in preclinical epithelial tumor models and should be distinguished from clinical studies of topical calcipotriol or high-dose oral vitamin D [77].

This mechanism has important implications for PDT selectivity. One of the major advantages of PDT is that cytotoxicity is spatially and chemically restricted: injury occurs primarily where the photosensitizer is present and where light is delivered.

The comparison between vitamin D-related and vitamin A-related interventions illustrates that biologically active compounds may affect PDT at different mechanistic levels. Selected vitamin A-related compounds may modify ROS-dependent injury, whereas calcitriol or vitamin D analogues may act upstream by altering PpIX biosynthesis. However, neither group should be assigned a universal directional effect, because outcomes depend on the individual compound and PDT system. The relevant criterion is not whether a vitamin has a general anticancer effect, but whether its dominant mechanism is compatible with the photochemical requirements of PDT.

Clinical investigation of vitamin D-related pretreatment has included distinct interventions that should not be combined into a single evidence category. Topical calcipotriol has been investigated before MAL-PDT in actinic keratoses, whereas high-dose oral vitamin D has been evaluated before ALA-PDT in basal cell carcinoma [68,78]. These interventions differ in compound, route of administration, lesion type, photosensitizer precursor, and clinical endpoint and should therefore be interpreted separately.

These clinical contexts are important because non-melanoma skin cancers and actinic keratoses are established indications for ALA- or MAL-based PDT. These lesions are superficial, accessible to topical drug application, and suitable for controlled illumination. They also allow direct monitoring of PpIX fluorescence, which provides a practical biomarker of photosensitizer accumulation. If vitamin D pretreatment increases lesion-specific PpIX fluorescence, this can be interpreted as a mechanistically meaningful enhancement of PDT readiness. For topical calcipotriol, the intervention is intended as a local pretreatment, whereas high-dose oral vitamin D represents a systemic intervention with a distinct pharmacological and clinical context.

The potential value of vitamin D pretreatment should nevertheless be interpreted with appropriate boundaries. The strongest mechanistic rationale applies to ALA/MAL-PDT, where PpIX biosynthesis is central to treatment. It should not automatically be generalized to all photosensitizer systems, especially those using exogenous photosensitizers whose accumulation does not depend on the heme biosynthetic pathway. Similarly, the clearest clinical relevance currently concerns superficial and accessible lesions, particularly non-melanoma skin cancers and premalignant keratinocytic lesions. Extension to deeper tumors or internal malignancies would require careful evaluation of delivery route, dosing, timing, tumor biology, and safety.

From a practical perspective, vitamin D illustrates how a neoadjuvant can be selected rationally for PDT. The broader implication is that PDT combination design should be based on pathway compatibility. ALA/MAL-PDT depends on heme biosynthesis, PpIX accumulation, oxygen availability, light activation, and ROS-mediated cytotoxicity. Calcitriol and selected vitamin D analogues may support this sequence at the level of PpIX production, whereas individual vitamin A-related compounds may modify ROS-mediated injury in either an attenuating or enhancing direction depending on the experimental context. This comparison reinforces the central argument of this article: biologically active compounds should be evaluated according to their interaction with the specific mechanism of PDT, not according to general assumptions about vitamins, supplements, or anticancer activity.

## 6. Clinical Applications of PDT in Oncology

Photodynamic therapy has found its strongest clinical position in tumors and premalignant lesions that are superficial, localized, or anatomically accessible to controlled light delivery. Its clinical usefulness results from the same three-component mechanism that defines its biological selectivity: photosensitizer accumulation, irradiation with an appropriate wavelength, and oxygen-dependent generation of reactive oxygen species. Because the cytotoxic effect is restricted mainly to illuminated tissue, PDT is particularly attractive in settings where local control, tissue preservation, cosmetic outcome, and reduced systemic toxicity are important therapeutic priorities.

The most established oncological indications for PDT involve non-melanoma skin cancers and premalignant keratinocytic lesions, including actinic keratoses, Bowen’s disease, and superficial basal cell carcinoma. In these conditions, ALA-PDT and MAL-PDT are especially valuable because the photosensitizing compound, protoporphyrin IX, is generated intracellularly after topical application of 5-aminolevulinic acid or methyl aminolevulinate. These lesions are accessible to direct illumination, and photosensitizer application can be limited to the diseased area. As a result, PDT can provide good local response rates while preserving surrounding tissue and producing favorable cosmetic outcomes compared with more destructive local approaches [79].

In actinic keratoses and Bowen’s disease, PDT is used both therapeutically and as part of field-directed treatment. This is clinically relevant because sun-damaged skin often contains multiple visible and subclinical premalignant changes within the same anatomical field. ALA/MAL-PDT can target these broader areas of dysplasia while avoiding extensive surgical excision. In superficial basal cell carcinoma, MAL-PDT is considered a tissue-sparing alternative in selected patients, particularly when surgery may lead to functional or cosmetic impairment. However, lesion thickness, histological subtype, localization, and recurrence risk remain important determinants of patient selection [80].

PDT has also been applied in selected gastrointestinal malignancies, particularly esophageal cancer and premalignant or early neoplastic lesions of the esophagus. In this setting, light can be delivered endoscopically, allowing treatment of mucosal or superficial lesions that are accessible from the lumen. Porfimer sodium-based PDT has historically been used for palliation of obstructive esophageal tumors and, in selected early-stage cases, with curative intent. The value of PDT in esophageal disease lies in its ability to ablate abnormal tissue while limiting systemic toxicity, although careful patient selection is essential because deeper or bulky tumors are less suitable for light-based therapy.

Endobronchial lung cancers represent another important group of tumors in which PDT can be clinically useful. When tumors are located within the bronchial tree and are accessible by bronchoscopy, light can be delivered directly to the lesion through fiber-optic systems. In obstructive endobronchial disease, PDT may provide palliative debulking, improve airway patency, and reduce tumor burden. In carefully selected early central lung cancers, PDT may also be used with curative intent. Its effectiveness in this context depends on tumor depth, bronchoscopic accessibility, oxygenation, and the ability to illuminate the entire target volume [81].

PDT has also been investigated in cholangiocarcinoma, particularly in combination with biliary stenting. Earlier studies suggested that endoscopic or percutaneous PDT could improve biliary drainage, reduce local tumor obstruction, and potentially improve clinical outcomes in patients with unresectable disease. However, these findings were not confirmed by the phase III PHOTOSTENT-02 trial, which reported inferior overall survival with stenting plus PDT compared with stenting alone. PDT should therefore be regarded as investigational and not routinely recommended for cholangiocarcinoma. Technical feasibility, tumor extent, light distribution, and photosensitizer pharmacokinetics remain important determinants of treatment delivery [82,83].

Beyond these established or better-documented uses, PDT is being investigated in bladder, prostate, and head-and-neck malignancies. These tumor types are attractive for PDT because they may be accessible through endoscopic, interstitial, intraoperative, or image-guided light delivery. In bladder cancer, intraluminal access creates a theoretical opportunity for treating superficial or multifocal disease. In prostate cancer, interstitial light delivery has been explored as a way to treat localized lesions while sparing surrounding structures. In head-and-neck cancers, PDT may be valuable for selected superficial, recurrent, or anatomically complex lesions where conventional surgery or re-irradiation carries significant morbidity. These applications remain more dependent on specialized protocols, photosensitizer selection, and precise dosimetry than dermatological PDT. The main established and investigational oncological applications of PDT are summarized in Table 2.

The clinical success of PDT depends strongly on anatomical accessibility. Superficial skin lesions are ideal because both photosensitizer delivery and illumination are straightforward. Endoscopic tumors, such as those in the esophagus, bronchial tree, bladder, and biliary tract, are also suitable when fiber-optic light delivery can reach the target. Deeper solid tumors are more challenging because visible and near-infrared light penetrate tissue only to a limited depth. This physical limitation remains one of the major barriers to expanding PDT into large, deeply seated, or poorly accessible malignancies.

Light penetration is closely related to the wavelength used. Longer wavelengths in the red or near-infrared range penetrate more deeply than shorter wavelengths, which is why second- and third-generation photosensitizers are often designed to absorb within the therapeutic optical window. However, even with improved photosensitizers, light attenuation by tissue scattering and absorption restricts the effective treatment depth. Photosensitizers activated within the red or near-infrared spectral range are being investigated as one approach to improving light penetration and extending PDT beyond superficial disease. Interstitial light delivery, intraoperative illumination, and two-photon or upconversion-based approaches are also being explored, although each requires separate supporting evidence [84].

Heterogeneous photosensitizer uptake also affects clinical response. Tumor tissue is biologically variable, and photosensitizer accumulation may differ between regions of the same lesion. Areas with insufficient photosensitizer concentration may receive light but fail to generate adequate ROS, leading to incomplete tumor destruction. Conversely, photosensitizer retention in normal tissues may increase off-target phototoxicity. This problem is especially important in internal malignancies, where direct monitoring of photosensitizer distribution is more difficult than in skin lesions. Fluorescence-guided assessment of PpIX accumulation, pharmacokinetic optimization, and targeted delivery systems are therefore important strategies for improving treatment uniformity [86].

Despite these limitations, PDT remains clinically valuable because it offers a unique combination of local selectivity, repeatability, tissue preservation, and compatibility with other treatments. It can be repeated in selected cases, integrated with surgery or endoscopic procedures, and combined with pharmacological or immunological strategies. Its role is strongest when the target lesion is accessible, sufficiently oxygenated, and capable of accumulating an effective photosensitizer concentration. Future clinical expansion will depend on improved photosensitizers, better light-delivery systems, real-time dosimetry, oxygen management, and rational combination approaches designed to overcome the biological and physical barriers that currently limit PDT efficacy.

## 7. Nanotechnology and Modern Photosensitizer Delivery Systems

Nanotechnology has become one of the most important directions in the development of modern photodynamic therapy because it addresses several limitations of classical photosensitizers at the same time. Many photosensitizers are hydrophobic, chemically unstable in biological fluids, prone to aggregation, and insufficiently selective for tumor tissue. These properties can reduce photodynamic efficiency by limiting bioavailability, promoting self-quenching, increasing off-target accumulation, and producing heterogeneous distribution within the tumor. Nanocarrier-based delivery systems are designed to improve solubility, protect photosensitizers from premature degradation, enhance tumor accumulation, enable controlled release, and support combination strategies with chemotherapeutic, immunological, or oxygen-generating agents.

The rationale for nanotechnology in PDT is closely linked to tumor biology. Solid tumors often possess abnormal vasculature, increased vascular permeability, impaired lymphatic drainage, acidic extracellular pH, hypoxic regions, and heterogeneous receptor expression. Nanocarriers can exploit some of these features to increase local drug accumulation. Passive targeting may occur through the enhanced permeability and retention effect, while active targeting can be achieved by attaching ligands such as antibodies, peptides, folic acid, or aptamers to the nanocarrier surface. In this way, the photosensitizer is no longer administered simply as a free light-sensitive molecule but as part of a delivery system designed to improve pharmacokinetics, selectivity, and therapeutic coordination [85].

Liposomal and lipid-based formulations are among the most established nanocarrier strategies for PDT. Liposomes can encapsulate hydrophobic photosensitizers within lipid bilayers or carry more hydrophilic compounds in their aqueous core. This improves dispersibility in biological fluids and may reduce nonspecific aggregation. Lipid-based systems can also modify biodistribution, limit premature clearance, and reduce accumulation in normal tissues responsible for prolonged photosensitivity. In PDT, these properties are particularly valuable because the photodynamic effect depends on achieving sufficient photosensitizer concentration in the target lesion while minimizing off-target retention. Liposomal systems may also be engineered for pH-responsive release, ligand-mediated targeting, or co-delivery with other therapeutic agents [87].

Polymeric nanoparticles represent another major class of photosensitizer delivery platforms. Materials such as poly(lactic-co-glycolic acid), polyethylene glycol-containing polymers, and other biodegradable polymeric systems can encapsulate photosensitizers and release them in a controlled or stimulus-responsive manner. Polymeric nanoparticles may improve the stability of hydrophobic photosensitizers, prolong circulation time, reduce systemic toxicity, and increase tumor accumulation. Their structure can be modified to respond to features of the tumor microenvironment, including acidic pH, redox gradients, enzymatic activity, or hypoxia. This enables more selective release of the photosensitizer within tumor tissue and reduces premature exposure of normal organs [88].

Gold nanoparticles and other inorganic nanostructures offer additional possibilities because they may combine photodynamic and photothermal mechanisms. Gold nanoparticles can absorb light and convert it into heat through plasmonic effects, producing localized hyperthermia. When combined with a photosensitizer, this creates the possibility of dual photodynamic and photothermal therapy under coordinated irradiation. The photothermal effect may damage tumor cells directly, improve membrane permeability, enhance drug release, or sensitize tissue to ROS-mediated injury. Such hybrid systems are particularly attractive for tumors that are partially resistant to PDT alone, although their clinical translation requires careful evaluation of biodistribution, long-term retention, toxicity, and light-delivery parameters [89].

Co-delivery platforms are among the most conceptually important advances in nanotechnology-based PDT. These systems allow a photosensitizer and a second therapeutic agent to be delivered together within a single carrier. The co-administered agent may be a chemotherapeutic drug, an immunomodulator, a molecular inhibitor, an oxygen-generating compound, or a molecule designed to reduce antioxidant defenses. The advantage of this approach is pharmacological coordination: both agents can reach the same tumor region at similar times and concentrations, increasing the likelihood of true therapeutic interaction [90]. In chemo-PDT, for example, a cytotoxic drug may damage DNA or disrupt proliferation while the photosensitizer generates ROS after irradiation. In immuno-PDT, the nanocarrier may combine local tumor destruction with immune stimulation, enhancing antigen release, dendritic cell activation, and T-cell priming.

Tumor hypoxia is one of the major obstacles to effective PDT, and nanotechnology offers several strategies to address it. Oxygen-generating nanoparticles can be designed to produce oxygen locally, for example, by decomposing endogenous hydrogen peroxide within the tumor microenvironment. Catalase-containing systems are one example of this approach, as they can convert hydrogen peroxide into oxygen and water, thereby increasing local oxygen availability during irradiation. Other strategies include oxygen-carrying nanocarriers, hypoxia-responsive drug release, vascular normalization approaches, and combination with agents that reduce oxygen consumption. By improving oxygen availability or adapting treatment to hypoxic conditions, these platforms aim to restore PDT efficacy in resistant tumor regions [91].

Nanotechnology is also important for natural and plant-derived photosensitizers. Compounds such as curcumin, hypericin, chlorophyll derivatives, pheophorbide derivatives, riboflavin, and berberine may have favorable biological properties and photosensitizing potential, but many suffer from poor aqueous solubility, low bioavailability, rapid metabolism, or limited tissue penetration [92]. Nanocarriers can improve their formulation and delivery by increasing solubility, protecting them from degradation, enhancing tumor uptake, and enabling controlled release. Carrier-free self-assembled nanostructures based on natural photosensitizers are especially attractive because they may achieve high photosensitizer loading while reducing the need for synthetic excipients [93].

The value of nanotechnology in PDT therefore extends beyond simple drug delivery. It can improve the physicochemical behavior of photosensitizers, increase tumor selectivity, reduce off-target toxicity, coordinate combination treatment, and address biological barriers such as hypoxia and heterogeneous uptake. These advantages are particularly relevant for expanding PDT beyond superficial lesions and improving its performance in solid tumors with complex microenvironments. However, nanotechnology-based PDT also introduces new challenges, including manufacturing reproducibility, long-term safety, regulatory complexity, pharmacokinetic variability, and the need for standardized evaluation of light dose, drug dose, and release kinetics.

**Figure 4:**
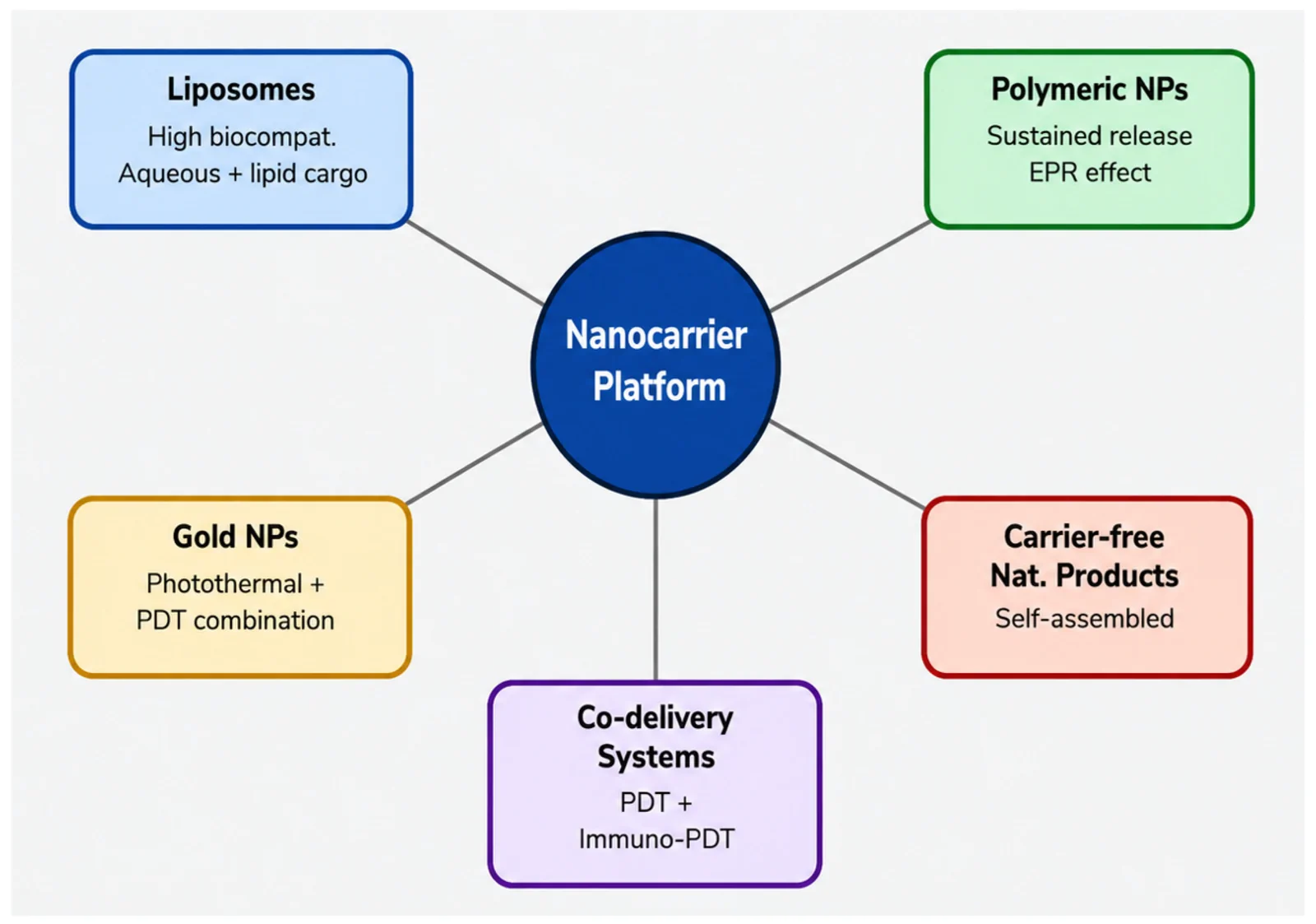
Nanotechnology Platforms in Photodynamic Therapy. Nanocarrier systems, including liposomes, polymeric nanoparticles, gold nanoparticles, co-delivery platforms, and carrier-free natural-product assemblies, can improve photosensitizer solubility, biocompatibility, controlled release, and combination delivery. Polymeric nanoparticles may provide sustained release and enhanced permeability and retention effects, while gold nanoparticles can support photothermal–PDT combinations. Co-delivery systems may enable PDT-based combination strategies, including immuno-PDT.

The future of nanotechnology-assisted PDT will depend on the development of platforms that are not only effective in preclinical models but also clinically practical. The most promising systems are likely to be those that solve a specific biological problem: poor photosensitizer solubility, inadequate tumor accumulation, hypoxia, uncontrolled release, or lack of immune activation. Rational design is therefore essential. A nanocarrier should not simply carry a photosensitizer; it should enhance the precise mechanism required for PDT success, whether by increasing photosensitizer loading, improving oxygen supply, enabling co-delivery, or amplifying antitumor immunity. The major nanotechnology-based strategies that address these limitations are summarized in Figure 4.

## 8. Photodynamic Therapy in Combination Treatment

Photodynamic therapy is increasingly considered not only as a local treatment modality, but also as a component of rational combination treatment. Its biological effects extend beyond direct tumor cell destruction and include oxidative damage, vascular shutdown, inflammatory signaling, and immunogenic cell death. These mechanisms create opportunities for combining PDT with chemotherapy, radiotherapy, immunotherapy, nanotechnology-based delivery systems, and selected biological modulators. However, PDT combinations must be designed carefully. The objective is not simply to add another anticancer agent but to select agents that reinforce the photodynamic mechanism rather than suppress it.

The combination of PDT with chemotherapy is based on the possibility of complementary or synergistic tumor injury. Chemotherapeutic agents may damage DNA, inhibit cell division, impair repair pathways, disrupt metabolism, or sensitize tumor cells to oxidative stress. PDT, in turn, produces spatially controlled ROS-mediated cytotoxicity after activation of a photosensitizer by light. In selected settings, chemotherapy may also increase the accumulation of photosensitizing intermediates. For example, methotrexate and 5-fluorouracil have been investigated as enhancers of ALA-PDT because they may increase intracellular protoporphyrin IX accumulation and sensitize tumor cells before light exposure. In such combinations, chemotherapy does not merely act as an additional cytotoxic modality; it may prepare tumor cells for a stronger photodynamic response [94,95].

Chemo-PDT is particularly attractive because it combines systemic or regional pharmacological activity with the local selectivity of light activation. A cytotoxic drug may weaken tumor cell survival mechanisms or increase vulnerability to oxidative injury, while PDT can destroy cells in the illuminated region through ROS generation. Nanotechnology-based co-delivery platforms further improve this concept by allowing a photosensitizer and a chemotherapeutic drug to be carried within the same delivery system. This may reduce pharmacokinetic mismatch, improve tumor accumulation, and increase the probability that both agents reach the same tumor region at therapeutically relevant concentrations. Nevertheless, chemo-PDT requires careful optimization of dose, timing, drug-light interval, and sequence of administration, because an improperly chosen partner may fail to enhance PDT or may even interfere with its oxidative mechanism [85,90,93].

The relationship between PDT and radiotherapy is more complex because both treatments can involve oxidative damage. Radiotherapy induces tumor injury through direct DNA damage and through indirect mechanisms involving water radiolysis and ROS formation. PDT induces localized oxidative injury through light-activated photosensitizers. In principle, these modalities may complement each other because radiotherapy can reach deeper tumor volumes, while PDT can intensify local treatment in superficial, mucosal, or endoscopically accessible lesions. This combination may be relevant in selected clinical situations where local recurrence or residual disease remains accessible to light delivery [96].

However, the shared dependence on oxidative stress also introduces important limitations. If PDT and radiotherapy are combined or sequenced, clinicians must consider cumulative tissue injury, inflammation, oxygen depletion, vascular damage, wound healing, and normal tissue tolerance. Moreover, redox-modifying agents used during either therapy require caution. High-dose supplementation with specific vitamin A-related compounds, including β-carotene, may theoretically modify ROS-mediated injury; however, the direction and magnitude of this effect are likely to depend on the compound, dose, treatment modality, and experimental or clinical context. Therefore, the compatibility of supportive agents and supplements must be evaluated according to mechanism rather than assumed benefit.

Among combination strategies, PDT combined with immunotherapy is one of the most promising. PDT can induce immunogenic cell death, characterized by the exposure or release of signals such as calreticulin, ATP, HMGB1, and tumor-associated antigens. These signals can recruit and activate antigen-presenting cells, promote dendritic cell maturation, and support the priming of tumor-specific cytotoxic T lymphocytes. In this way, local photodynamic injury may generate broader antitumor immune responses [30,97]. PDT may therefore function not only as a cytotoxic treatment, but also as an immune-activating intervention capable of converting tumor destruction into an antitumor immune response.

Immune checkpoint inhibitors are particularly rational partners for PDT. Antibodies targeting PD-1, PD-L1, or CTLA-4 can reduce inhibitory signaling that suppresses T-cell function in the tumor microenvironment. PDT may provide the antigenic and inflammatory stimulus, while checkpoint blockade may sustain and amplify the resulting T-cell response. The biological sequence is coherent: PDT damages tumor cells and releases antigens; antigen-presenting cells process and present these antigens; T cells become activated; checkpoint inhibitors prevent premature functional suppression of the antitumor immune response. Preclinical studies indicate that combining PDT with immune checkpoint blockade can improve local tumor control and may contribute to systemic antitumor effects, including regression of distant non-irradiated lesions in selected experimental models [98].

The concept of PDT as a local antitumor vaccine is central to immuno-PDT [99]. Unlike conventional vaccination, PDT does not introduce a predefined antigen. Instead, it causes tumor cells to release their own antigenic repertoire in an inflammatory context. This may be particularly valuable in heterogeneous tumors, where multiple tumor-associated antigens may coexist. By producing local oxidative injury and immunogenic cell death, PDT can transform the treated tumor into an in situ source of antigenic material. If the immune response is adequately supported, this local event may contribute to broader antitumor immune surveillance.

Despite this potential, immuno-PDT remains dependent on treatment parameters and tumor context. Excessive tissue destruction may produce necrosis without optimal immune priming, whereas insufficient treatment may fail to release enough antigen or danger signals. The immunological phenotype of the tumor is also important. Inflamed tumors with pre-existing immune infiltration may respond differently from immune-excluded or immune-desert tumors. The type of photosensitizer, light dose, fluence rate, timing of immunotherapy, degree of vascular damage, and local cytokine profile may all influence whether PDT produces only local cytotoxicity or a durable systemic immune response.

The main PDT combination partners and their expected mechanistic interactions are summarized in Table 3.

Overall, PDT combination therapy should be guided by a mechanism-first approach. The strongest combinations are those that improve one of the limiting steps of PDT: photosensitizer delivery, PpIX accumulation, oxygen availability, vascular targeting, or immune activation. Potentially unfavorable combinations are those that interfere with these processes under the specific compound, dose, photosensitizer, tumor model, and treatment conditions evaluated. This principle is directly relevant to clinical practice because patients may assume that all supplements, vitamins, or antioxidants are beneficial during cancer treatment. In the specific context of PDT, this assumption may be incorrect. Combination therapy should therefore be based on photochemical and biological compatibility, not on general therapeutic optimism.

## 9. Limitations of Current Evidence and Future Directions

Although photodynamic therapy has a strong mechanistic foundation and established clinical value in selected oncological indications, several limitations continue to restrict its broader translation and optimization [100]. These limitations concern not only the technology of PDT itself, but also the evidence base supporting specific combination strategies, including the use of vitamin A, vitamin D, nanotechnology platforms, and immunotherapy. A critical interpretation of the available data is therefore essential. PDT is a promising modality, but its future development requires stronger experimental validation, better clinical standardization, and carefully designed trials that test mechanism-based combinations rather than empirically selected adjuncts.

A major limitation in PDT research is the lack of standardization across treatment parameters. PDT efficacy depends on photosensitizer dose, route of administration, drug-light interval, wavelength, light fluence, fluence rate, tissue oxygenation, lesion thickness, and tumor biology. Variability in any of these parameters can substantially alter treatment response. This makes it difficult to compare results across studies, institutions, photosensitizers, and tumor types. Standardization is especially important for multicenter clinical trials, where reproducibility depends on consistent dosimetry and clearly defined treatment protocols. Without standardized parameters, even promising combinations may appear inconsistent because differences in light delivery or photosensitizer pharmacokinetics obscure the true biological effect.

Light dosimetry remains one of the central technical challenges. The delivered light dose at the tissue surface does not necessarily correspond to the effective light dose received throughout the tumor volume. Tissue scattering, absorption, pigmentation, blood content, anatomical geometry, and tumor thickness all influence light distribution. Deeper tumor regions may receive insufficient fluence, leading to incomplete ROS generation even when the photosensitizer is present. Real-time dosimetry, fluorescence monitoring, oxygen monitoring, and image-guided light delivery may help address this problem. Future PDT protocols should increasingly incorporate measurable treatment parameters rather than relying solely on nominal light dose [35,36].

The development of targeted photosensitizers is another major direction for future research. Classical photosensitizers often suffer from limited tumor selectivity, off-target accumulation, prolonged photosensitivity, hydrophobicity, aggregation, and heterogeneous tumor uptake. Third-generation photosensitizers attempt to overcome these limitations through ligand targeting, antibody conjugation, peptide-based delivery, folate receptor targeting, aptamers, and nanocarrier systems. The goal is not only to increase tumor accumulation, but also to improve intracellular localization, reduce normal tissue toxicity, shorten post-treatment photosensitivity, and enable combination treatment. Future photosensitizers should be evaluated not merely by absorption spectra and singlet oxygen yield, but by pharmacokinetics, tumor-to-normal tissue ratios, intracellular localization, immune effects, and compatibility with clinical light-delivery systems [101].

Nanotechnology-based PDT also requires further translational work. Many nanocarrier systems show strong preclinical promise, including liposomes, polymeric nanoparticles, gold nanoparticles, oxygen-generating platforms, and carrier-free natural-product assemblies. These systems can improve solubility, tumor accumulation, controlled release, hypoxia mitigation, and co-delivery of synergistic agents. However, clinical translation remains challenging because nanomedicines introduce complexity in manufacturing, stability, batch reproducibility, biodistribution, long-term safety, clearance, regulatory evaluation, and cost. Future research should prioritize platforms that solve clearly defined clinical problems rather than adding technological complexity without a specific therapeutic advantage [102].

The combination of PDT with immunotherapy represents another promising but still developing field. PDT-induced immunogenic cell death can release tumor antigens and danger signals capable of stimulating dendritic cell activation and T-cell priming. Immune checkpoint inhibitors may amplify this response by preventing T-cell exhaustion or suppression. However, the optimal sequencing, dose, photosensitizer choice, light parameters, and immunotherapy timing remain unresolved. Future studies should evaluate not only local tumor response, but also systemic immune activation, T-cell infiltration, antigen presentation, cytokine profiles, distant lesion control, and durable memory responses. Clinical translation will require careful selection of tumor types most likely to benefit from immune activation, such as immunologically inflamed or locally accessible tumors [103].

A broader limitation is that PDT has historically been underrepresented in large randomized clinical trials compared with surgery, radiotherapy, systemic chemotherapy, and modern targeted therapies. This is partly due to practical challenges: PDT requires specialized photosensitizers, light-delivery systems, dosimetry expertise, and procedural coordination. It is also difficult to standardize across tumor sites because each anatomical location requires different methods of photosensitizer administration and illumination. To establish PDT more firmly within multimodal oncology, future research should include well-designed comparative trials, standardized endpoints, long-term follow-up, and integration with modern imaging and biomarker strategies.

Future studies should also examine patient behavior, especially supplement use during cancer treatment. Because patients may take vitamin A, β-carotene, antioxidant combinations, herbal products, or vitamin D without informing clinicians, supplement exposure may act as an uncontrolled confounder in PDT outcomes. Clinical trials should document supplement use systematically, and observational studies should evaluate whether specific antioxidant or retinoid interventions are associated with altered responses to PDT or other oxidative therapies, including both attenuation and enhancement. At the same time, supervised use of vitamin D analogues as PDT pretreatment should be distinguished from unsupervised systemic supplementation. This distinction is crucial for translating mechanism-based guidance into safe clinical practice.

Overall, the future of PDT depends on moving from empirical combinations toward rational, mechanism-driven treatment design. The most important priorities are determining in vivo whether specific vitamin A-related compounds attenuate, enhance, or do not alter PDT under defined experimental conditions; standardizing PDT dosimetry and reporting; developing targeted and clinically practical photosensitizers; translating nanotechnology platforms with clear therapeutic advantages; optimizing vitamin D-enhanced ALA/MAL-PDT; and defining the role of PDT in immunotherapy-based regimens. These directions will determine whether PDT remains primarily a local treatment for selected accessible lesions or becomes a broader platform for integrated oncological therapy.

## 10. Conclusions

PDT is an anticancer treatment whose effectiveness depends on the precise interaction between a photosensitizer, light of the appropriate wavelength, and molecular oxygen in the target tissue. Upon activation, the photosensitizer generates reactive oxygen species (ROS), which induce direct tumor cell death, vascular damage, and immunogenic cell death. This makes PDT a biologically integrated therapeutic platform rather than a purely localized light-based approach. Because PDT relies on the generation of ROS, the selection of adjuvants should be guided by their compatibility with the underlying photodynamic mechanism. Compounds capable of increasing photosensitizer accumulation, improving oxygen availability, enhancing oxidative damage, or promoting antitumor immune responses can improve treatment efficacy. Conversely, agents that interfere with these processes can potentially alter treatment outcomes. Therefore, when designing combination strategies involving PDT, mechanistic compatibility, rather than general pharmacological properties, should be considered. Among the vitamin-based approaches discussed in this review, vitamin D-related interventions have shown potential as adjuvants to ALA- and MAL-based PDT for superficial keratinocytic lesions and non-melanoma skin cancers. However, the available evidence concerns distinct interventions that should be interpreted separately: preclinical calcitriol pretreatment in epithelial tumor models, topical calcipotriol before MAL-PDT in actinic keratoses, and high-dose oral vitamin D before ALA-PDT in basal cell carcinoma. These approaches differ in the compound used, route of administration, lesion type, photosensitizer precursor, and study endpoint. Collectively, the findings suggest that selected vitamin D-related interventions may increase intracellular protoporphyrin IX accumulation or improve treatment response under defined conditions. However, evidence supporting similar effects in other photosensitizer systems or cancer types remains limited. The interaction between vitamin A derivatives and PDT, however, remains incompletely understood. Although some retinoids possess antioxidant and cytoprotective properties that could theoretically influence ROS-dependent photodynamic mechanisms, the available experimental evidence is limited, heterogeneous, and largely confined to preclinical models. Current data do not support the conclusion that vitamin A or its derivatives universally antagonize PDT, and further well-designed in vivo and clinical studies are necessary before clinically relevant recommendations can be made regarding vitamin A supplementation during PDT. Despite significant progress, the broader clinical application of PDT remains limited by several challenges, including limited light penetration, tumor hypoxia, heterogeneous photosensitizer distribution, lack of standardized optical dosimetry, and limited availability of large, well-controlled clinical trials. Continued advances in targeted photosensitizers, nanotechnology-based drug delivery systems, oxygen-modulating approaches, fluorescence-guided treatment monitoring, standardized dosimetry protocols, and combinations with immunotherapy are expected to further expand the therapeutic potential of PDT.

In summary, PDT should be considered a biologically integrated, ROS-dependent therapeutic platform whose success depends on the coordinated optimization of photosensitizer pharmacology, light delivery, oxygen availability, and rationally selected combination therapies. Future progress will rely on the development of mechanism-based treatment strategies supported by solid preclinical evidence and well-designed clinical trials, ultimately enabling more personalized and effective applications of photodynamic therapy in oncology.

## Figures and Tables

**Table 1:** Key optical dosimetric parameters influencing photodynamic therapy efficacy.

Parameter	Clinical Relevance
Wavelength (nm)	Determines photosensitizer activation efficiency and tissue penetration depth. Selection of an appropriate wavelength is essential for maximizing light absorption while ensuring adequate photon delivery to the target tissue.
Fluence (J/cm^2^)	Represents the total optical energy delivered per unit area. Fluence directly influences the cumulative photodynamic dose and the amount of ROS generated during treatment.
Fluence rate (mW/cm^2^)	Determines the rate of energy delivery. Excessively high irradiance may accelerate oxygen depletion and reduce ROS production, whereas lower irradiance may improve tissue reoxygenation and treatment efficacy.
Exposure time	Defines the duration of illumination. Together with irradiance, it determines the total delivered fluence and influences oxygen replenishment during PDT.
Light source	Lasers provide monochromatic, highly controllable illumination, whereas LEDs offer broader illumination fields and lower cost. Daylight PDT represents an alternative approach for selected superficial lesions.
Tissue optical properties	Tissue absorption, scattering, vascularization, pigmentation, and anatomical structure determine photon distribution, penetration depth, and the effective light dose reaching the photosensitizer.

**Table 2:** Approved and Investigational Clinical Indications for PDT Across Tumor Types.

Indication/Lesion Type	PDT Modality	Clinical Status/Key Evidence
Actinic keratoses/Bowen’s disease	ALA-PDT/MAL-PDT	Established treatment option for selected lesions; high complete response rates and favorable cosmetic outcomes [2,79,80].
Superficial basal cell carcinoma	MAL-PDT	Established alternative to surgery for appropriately selected low-risk superficial lesions; favorable cosmetic outcomes [2,79].
Esophageal cancer/endobronchial non-small-cell lung cancer	Porfimer sodium (Photofrin)-PDT	Established clinical application in selected settings; used for early-stage lesions and palliative management of obstructive disease [3,81].
Cholangiocarcinoma	Porfimer sodium (Photofrin)-PDT (endoscopic)	Investigational; not routinely recommended. Early studies suggested potential benefit from biliary stenting plus PDT; however, the phase III PHOTOSTENT-02 trial reported inferior overall survival compared with stenting alone [82,83].
Bladder cancer	Second- and third-generation photosensitizers	Investigational application; further clinical evaluation is required [84].
Prostate cancer	ALA-PDT/second-generation photosensitizers	Preclinical evidence supports further investigation of ALA-PDT in prostate cancer [17].
Head and neck cancer	Second- and third-generation photosensitizers	Investigational; evidence from oral squamous cell carcinoma supports further clinical evaluation [85].
Actinic keratoses + vitamin D pretreatment	Calcipotriol + MAL-PDT	Randomized clinical trial evidence indicates increased PpIX accumulation and improved lesion clearance compared with MAL-PDT alone [78].

**Table 3:** Summary of PDT Combination Agents and Mechanistic Interactions.

Agent	Proposed Mechanism of Interaction with PDT	Reported Effect on PDT	Highest Level of Evidence
Calcitriol	Modulation of differentiation and heme-biosynthetic enzymes, increasing PpIX accumulation before ALA-PDT	Enhancing under defined preclinical conditions	Preclinical evidence [77]
Topical calcipotriol	Local pretreatment before MAL-PDT may increase lesion-specific PpIX accumulation	Enhancing in actinic keratoses	Randomized clinical evidence [78]
High-dose oral vitamin D	Neoadjuvant administration before ALA-PDT	May accelerate lesion clearance in basal cell carcinoma	Randomized clinical evidence [68]
Vitamin A-related compounds	Compound-specific effects involving retinoid signaling, differentiation, antioxidant activity, membrane stabilization, or singlet-oxygen quenching	Context-dependent; attenuation and enhancement have both been reported	Preclinical evidence [17,18,34,60,74]
Methotrexate	DHFR inhibition and prodifferentiation effects may increase PpIX accumulation before ALA-PDT	Enhancing	Preclinical evidence [94]
5-Fluorouracil	Pretreatment may enhance porphyrin-pathway activity and PpIX accumulation before ALA/MAL-PDT	Enhancing	Clinical evidence in actinic keratoses [95]
Nanocarriers	Improved photosensitizer delivery, co-delivery of therapeutic agents, enhanced tumor accumulation, and mitigation of hypoxia	Enhancing in selected systems	Robust preclinical evidence; emerging clinical translation [45,86,87,88,89,90,91,92,93]
Immune checkpoint inhibitors	PDT-induced immunogenic cell death combined with PD-1/PD-L1 or CTLA-4 blockade may enhance systemic antitumor immunity	Enhancing in selected experimental models	Preclinical evidence [31,32,98]

Abbreviations: ALA, 5-aminolevulinic acid; DHFR, dihydrofolate reductase; PDT, photodynamic therapy; PpIX, protoporphyrin IX. Evidence categories were assigned descriptively according to the criteria stated in the Section 2. Preclinical evidence refers to in vitro or in vivo experimental studies; early clinical investigation refers to preliminary human studies without established clinical efficacy; and randomized clinical evidence refers to controlled randomized studies. These categories organize the narrative synthesis and do not constitute a formal evidence-grading system.

## Data Availability

No new data were created or analyzed in this study. Data sharing is not applicable to this article.
